# The Efficacy of Web-Based Cognitive Behavioral Therapy With a Shame-Specific Intervention for Social Anxiety Disorder: Randomized Controlled Trial

**DOI:** 10.2196/50535

**Published:** 2024-06-27

**Authors:** Xu Wen, Mengke Gou, Huijing Chen, Tomoko Kishimoto, Mingyi Qian, Jürgen Margraf, Thomas Berger

**Affiliations:** 1 Department of Clinical Psychology and Psychotherapy, Ruhr University Bochum, Bochum, Germany; 2 School of Psychological and Cognitive Science, Peking University, Beijing, China; 3 School of Sociology and Political Science, Shanghai University, Shanghai, China; 4 Department of Social Psychology, Nankai University School of Sociology, Tianjin, China; 5 Department of Psychology, Clinical Psychology and Psychotherapy, University of Bern, Bern, Switzerland

**Keywords:** social anxiety disorder, web-based cognitive behavioral therapy, shame intervention, mediating effects, shame experience

## Abstract

**Background:**

Social anxiety disorder (SAD) is one of the most prevalent psychological disorders and generally co-occurs with elevated shame levels. Previous shame-specific interventions could significantly improve outcomes in social anxiety treatments. Recent review suggests that integrating a more direct shame intervention could potentially increase the effectiveness of cognitive behavioral therapy. Web-based cognitive behavioral therapy (WCBT) has proven efficacy, sustaining benefits for 6 months to 4 years. Previous evidence indicated that shame predicted the reduction of social anxiety and mediated between engagements in exposure and changes in social anxiety during WCBT.

**Objective:**

This study aimed to design a shame intervention component through a longitudinal study and conduct a randomized controlled trial to investigate the effectiveness of a shame intervention component in reducing social anxiety symptoms and shame experience in a clinical sample of people with SAD.

**Methods:**

The development of a shame intervention component was informed by cognitive behavioral principles and insights from longitudinal data that measured the Experience of Shame Scale (ESS), the Coping Styles Questionnaire, and the Social Interaction Anxiety Scale (SIAS) in 153 participants. The psychoeducation, cognitive construct, and exposure sections were tailored to focus more on shame-related problem-solving and self-blame. A total of 1220 participants were recruited to complete questionnaires, including the ESS, the SIAS, the Social Phobia Scale (SPS), and diagnostic interviews. Following a 2-round screening process, 201 participants with SAD were randomly assigned into a shame WCBT group, a normal WCBT group, and a waiting group. After the 8-week WCBT intervention, the participants were asked to complete posttest evaluations, including the ESS, SIAS and SPS.

**Results:**

Participants in the shame WCBT group experienced significant reductions in shame levels after the intervention (ESS: *P*<.001; ηp^2^=0.22), and the reduction was greater in the shame intervention group compared to normal WCBT (*P*<.001; mean deviation −12.50). Participants in both the shame WCBT and normal WCBT groups experienced significant reductions in social anxiety symptoms (SIAS: *P*<.001; ηp^2^=0.32; SPS: *P*<.001; ηp^2^=0.19) compared to the waiting group after intervention. Furthermore, in the experience of social interaction anxiety (SIAS), the shame WCBT group showed a higher reduction compared to the normal WCBT group (*P*<.001; mean deviation −9.58). Problem-solving (SE 0.049, 95% CI 0.025-0.217) and self-blame (SE 0.082, 95% CI 0.024-0.339) mediated the effect between ESS and SIAS.

**Conclusions:**

This is the first study to design and incorporate a shame intervention component in WCBT and to validate its efficacy via a randomized controlled trial. The shame WCBT group showed a significant reduction in both shame and social anxiety after treatment compared to the normal WCBT and waiting groups. Problem-solving and self-blame mediated the effect of shame on social anxiety. In conclusion, this study supports previous findings that a direct shame-specific intervention component could enhance the efficacy of WCBT.

## Introduction

Social anxiety disorder (SAD), also known as social phobia, is characterized by an overwhelming fear of negative interpersonal evaluations and avoidance of embarrassing or shameful social situations [1], and it is one of the most common psychological disorders, with a 2% global prevalence [2]. The pathological model of SAD suggests that cognitive and behavioral patterns play an important role in the development and maintenance of social anxiety symptoms [3].

Previous SAD intervention research confirmed that socially anxious individuals generally have higher levels of shame [4-6], with more than half of patients with SAD reporting having experienced shame in situations of social anxiety [7,8]. A recent systematic review defined shame as a complicated experience including self-critical cognition, as well as safety and avoidance behavior [9], which shares similar manifestations with SAD [10,11]

Cognitive behavioral therapy (CBT) is considered the gold standard psychotherapy for SAD [12]. Several CBT framework group interventions with shame-specific interventions have showed favorable outcomes in enhancing the efficacy of SAD treatment in nonclinical samples. For example, Li et al [13] conducted cognitive behavioral group therapy (CBGT) among socially anxious individuals and found that CBGT with a shame intervention was significantly more effective than traditional CBT interventions; Golden [14] developed a shame CBGT program for social anxiety, adding the components of “acceptance of shame” and “exposure to shame” to the exposure component of the traditional CBT intervention, and successfully applied it to a group of university students. Therefore, given the consistent empirical evidence of a strong and robust positive relationship between shame and social anxiety [9,15,16], incorporating direct interventions for shame may also enhance the efficacy of CBT for treating SAD.

Web-based CBT (WCBT) has many significant advantages over face-to-face CBT, such as being less time-consuming, less costly, and easier to implement [17]. The effectiveness of WCBT is significant and can be maintained for 4 years after the end of the intervention [18-20]. In China, successive studies have confirmed that scores for social anxiety in WCBT intervention groups are significantly lower in posttests than pretests and that the efficacy persists for 6 months after the end of the intervention [6,21,22]. Furthermore, shame fully mediated the relationship between engagement in exposure and changes in social anxiety during the WCBT intervention [23]. However, no WCBT study has yet designed a component specifically for shame intervention, nor has any study investigated the efficacy of WCBT with a direct shame intervention in clinical patients with social anxiety.

Although shame-related thoughts (eg, “I’m annoying”) could benefit from WCBT interventions like restructuring cognitive biases to adaptive and flexible thoughts [6,21], direct evidence supporting the efficacy of shame WCBT interventions remains scarce. Interventions for social anxiety in Eastern cultural contexts should particularly address issues of shame, as individuals might “normalize” their socially anxious behaviors and emotions as experiences of shame [24,25]. Moreover, shame is noted to influence the severity of social anxiety and further sustain the symptoms of social anxiety development in Eastern cultures [13]. Other randomized controlled trials on shame also found that improvements in shame experience could predict the reduction of social anxiety and positive treatment results [26,27]. Therefore, incorporating shame interventions into WCBT holds promise for helping individuals with social anxiety effectively cope with shame and social anxiety.

Overall, the role of shame interventions in WCBT for SAD has been significantly underexplored. Recent evidence indicates that integrating a more direct shame intervention could potentially increase the effectiveness of CBT [9]. Despite WCBT for social anxiety possibly benefiting shame experiences, a WCBT intervention with a shame-specific intervention might be more effective. The objective of this study was to design a shame intervention component within WCBT and investigate its effectiveness. Initially, the relationship between shame, coping styles, and social anxiety was explored through a longitudinal study, forming the basis for designing a shame-specific intervention in WCBT. Subsequently, after 2 rounds of measurements and screenings, patients with social anxiety were randomized into a shame WCBT, a normal WCBT, and a waiting group. Following the 8-week WCBT interventions, the study assessed differences in social anxiety scores among the 3 groups to evaluate the efficacy of the shame WCBT. Given the high association of social anxiety and depression [25], changes in depression scores before and after treatment were also measured in this study.

Based on the above overview, we hypothesized the following: first, there would be a significant difference between the pre- and posttests of social anxiety and shame between the shame WCBT group and the normal WCBT group, and the efficacy in the shame WCBT group would be significantly higher than that in the normal group; that is, the Social Interaction Anxiety Scale (SIAS), the Social Phobia Scale (SPS), and the Experience of Shame Scale (ESS) scores of the participants in the shame WCBT group would be significantly lower than in the normal WCBT group. Second, there would be a significant difference between the pre- and posttests for social anxiety, shame, and depression between both intervention groups and the waiting group. That is, after the treatment, the shame WCBT group and the normal WCBT group would show a greater reduction in SIAS, SPS, and ESS scores compared to the waiting group. Third, the direct effect of shame experience on social anxiety in the longitudinal study would be significant, and several coping styles would significantly mediate the effect between shame and social anxiety.

## Methods

### The Process of Designing the Shame Intervention Component for WCBT

The WCBT used in this study was adapted from a WCBT program developed in Switzerland. The main intervention sessions were divided into 5 components: motivation and psychoeducation, cognitive reconstruction, attention training, exposure exercises, and problem-solving (details of the specific translation process and session content are described in previous publications [21]). Based on this WCBT program, the shame intervention components were designed and added to a longitudinal study that investigated the relationship between shame and social anxiety. First, to explore shame, coping behaviors, and their relationship to social anxiety, 153 participants were recruited on the web and provided informed consent. They first completed the ESS and the Coping Styles Questionnaire (CSQ). After 1 month, they were assessed with the SIAS. Demographic variables and the results of this part of the study are shown under the subheading Demographic Information and Analysis of Shame Intervention Design. This longitudinal study attempted to provide guidance for the shame intervention in line with the CBT framework, which focuses on self-critical thoughts, social avoidance, and anxious experience [28,29]. The adaptation of the shame WCBT program targeted the modification with 3 treatment sections and homework assignments. First, for the psychoeducation section, additional psychoeducation on shame was incorporated into the section on understanding the anxiety experience and anxiety disorders. Participants could learn and recognize the concept of shame and understand the relationship between shame and social anxiety. It was emphasized that inappropriate coping with shame can even exacerbate symptoms of social anxiety. The assigned homework comprised an analysis of the role of shame in the circle of social anxiety. Second, for the cognitive construct section, interventions targeted on negative perceptions of shame and attribution (self-blame coping) were added to the section on rational thinking. For homework, participants were asked to identify shame-related irrational thoughts and provide evidence. Third, for the exposure section, participants were encouraged to recognize their shame experiences, confront the shame-inducing situation, and solve problems (problem-solving coping), including not avoiding scenarios that may trigger shame. In the section on authenticity testing, shame-related exposure exercises were added as homework. After the content of the revised program was approved and updated on the website, 7 undergraduate students who had never been exposed to WCBT and had not studied CBT were recruited as pretest participants. They followed a strict process of accessing the website, studying the revised version of the program, providing comments and suggestions, and engaging in discussions.

### WCBT Intervention Recruitment and Screening Process

All the participants in the WCBT intervention were recruited through various online recruitment channels, including the official website, Weibo, WeChat, and other platforms. The first round of screening was based on the list described by Berger et al [30] of people who are suitable for project intervention: (1) a total SIAS score higher than 32 or a total SPS score higher than 22, (2) age 18 years or older, (3) no previous diagnosis of any mental illness or disorder, (4) no psychotropic medication within the last year, and (5) not having received any form of psychotherapy or psychological counseling. A total of 515 participants passed the first round of screening.

The second round of screening included diagnosed SAD patients and excluded participants who met the diagnostic criteria for certain types of psychiatric or psychological disorders. All participants who passed the first screening were invited to a one-on-one diagnostic interview. All interviewers were postgraduate or doctoral students in clinical psychology who were competent in the Mini-International Neuropsychiatric Interview (MINI) and were supervised by a licensed psychiatrist. At the end of the interview, a total of 201 participants met the following criteria and were able to enter the intervention period: (1) met the criteria in the MINI for SAD, (2) did not have a moderate or high risk of suicide, and (3) did not meet other diagnostic criteria, except major depression disorder.

**Figure 1.**
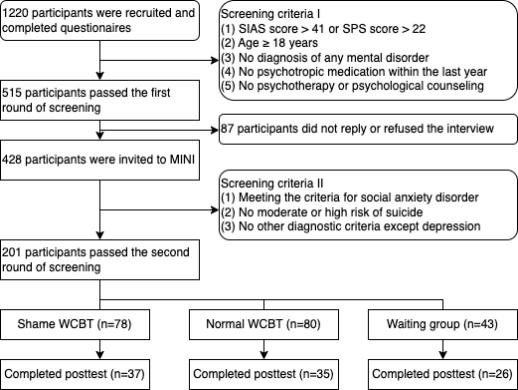
Flowchart of WCBT intervention screening process. MINI: Mini-International Neuropsychiatric Interview; SIAS: Social Interaction Anxiety Scale; SPS: Social Phobia Scale; WCBT: web-based cognitive behavioral therapy.

After 2 rounds of screening, a total of 201 participants were enrolled in the project and received the WCBT intervention for 8 consecutive weeks. Participants were asked to complete pre-and posttest questionnaires before and after the WCBT. Upon logging on to the website for the first time, the website automatically randomly assigned them to the shame group, normal group, or waiting group at a 2:2:1 ratio. The shame and normal groups started the intervention immediately, while the waiting group waited 8 weeks before starting it. The screening, grouping process, and completion status of all participants are shown in Figure 1.

### Materials and Measurement Tools

The Chinese versions of the SIAS and SPS were revised from the English versions developed by Mattick and Clarke [31,32]. The SIAS measures individuals’ levels of anxiety, fear, and worry in general social interaction situations, while the SPS assesses the level of anxiety, fear, and worry in situations where individuals are observed by others. Each item in both scales is scored on a 5-point scale from 0 (not at all) to 4 (completely). The scale has good reliability and validity, with an internal consistency coefficient of 0.862 for SIAS and 0.904 for SPS.

The MINI is recognized as a simple and valid definitive interview tool for screening and assessing the *Diagnostic and Statistical Manual of Mental Disorders, Fourth Edition* (*DSM-IV*) and the *International Statistical Classification of Mental Disorders* (*ICD-10*) [33,34]. The MINI has good reliability and validity.

The ESS examines individual shame in 3 areas: personality, behavior, and body. It consists of 25 questions [35]. Each item is rated on a scale from 1 (not at all) to 4 (often), with higher totals representing higher levels of shame. The scale has good reliability indicators, with internal consistency reliability of 0.825 and retest reliability of 0.88.

The CSQ classifies how individuals cope with difficulties in everyday situations in 6 dimensions: problem-solving, self-blame, help-seeking, fantasy, avoidance, and rationalization, with the number of items under each dimension varying from 4 to 9 [36]. All dimensions of the scale have good reliability and validity, with retest reliability ranging from 0.62 to 0.72. The results are relatively stable and reliable.

The Chinese version of the Beck Depression Inventory (BDI) was used to assess depression [37,38]. The internal consistency reliability of the BDI is 0.879, which indicates good structural validity.

### WCBT Intervention Procedure

Participants with SAD were recruited and screened to compare the efficacy of the 2 different WCBT interventions (the shame WCBT and normal WCBT) for SAD and their effects on shame and anxiety levels over a period of 8 weeks. During the 8-week intervention period, participants were able to repeat or practice the material provided on the web, and they could also revise their submitted assignments [6]. All course content was presented in text format with the possibility to attach pictures, except for the relaxation training section, which had audio guidance. The recommended pace of study was 1 session per week plus time to complete and submit assignments, with approximately 2 to 3 hours spent per week [18].

### Study Blinding, Data Processing, and Analysis

This study was conducted with a double-blind design. First, both participants and interviewers were unaware of the specifics of the shame intervention and group assignments. Blind assignments were achieved through the use of a computerized randomization process on the website, which automatically allocated participants to intervention groups. Finally, the data analysis phase was conducted on anonymized data sets, ensuring that researchers remained blinded to group allocations until the conclusion of the statistical analysis.

SPSS (version 24.0; IBM Corp) was used for statistical analysis of the data in this study. There was only 1 set of missing data, for the gender and age of 1 participant. We opted not to use imputation and proceeded with complete cases. Demographic information statistics and basic descriptive statistics were analyzed with an ANOVA in the first step. Comparative data were followed by an analysis of the differences in scores for SIAS, SPS, ESS, and BDI measured before and after the study period in the waiting group, normal WCBT group, and shame WCBT group with a repeated measures ANOVA. Longitudinal data from the intervention design used the process self-sampling procedure developed by Preacher and Hayes [39] to analyze the relationship between shame experiences, coping styles, and levels of social anxiety. The shame experience score was used as the independent variable, the 6 subscale scores for coping style as the mediating variables, the social interaction anxiety (from SIAS) score as the dependent variable, and gender and age as control variables; the sampling number was then set to 5000.

### Sample Size Estimation

The sample size calculation for this study was conducted using G*Power (version 3.1; Universität Düsseldorf) [40]. This study aimed to detect at least a medium effect size (Cohen *f*=0.25) in between-group and within-group effects across the 3 intervention groups. Based on previous WCBT studies, which reported a correlation of approximately 0.5 between pre- and posttest scores, the sample size calculation for the repeated measures ANOVA indicated that each intervention group would require a minimum of 50 participants to achieve a statistical power of at least 0.8 at a significance level of .05. Considering previous studies have consistently shown medium to large effect sizes (*d*>0.5) for WCBT interventions compared to waiting groups, the sample size of the waiting group was set at half of the intervention groups.

### Ethical Considerations

All participants provided informed consent before accessing the recruitment questionnaire. The informed consent form provided a detailed description of the purpose, content, screening process, possible benefits and risks, costs, and start and exit of the project. This study was approved by the Committee for Protecting Human and Animal Subjects, Department of Psychology, Peking University (20180504) and registered at Peking University. The trial registration number (Chinese Clinical Trial Registry) is ChiCTR2300072184. All data were anonymized during the data processing and analysis. No monetary compensation or fees were provided. Participation in the WCBT intervention itself was considered compensation for participants.

## Results

### Pre- and Posttest Scores Among Patients With SAD

A total of 201 participants successfully entered the WCBT intervention program after 2 rounds of screening, with an average age of 27.8 (SD 6.82) years; 67.7% (n=136) were female. According to automatic random assignment, 78 participants (mean age 26.6, SD 5.38 years; n=57, 73% female) were assigned to the shame WCBT group, 80 participants (mean age 28.1, SD 7.04 years; n=51, 65% female) were assigned to the normal WCBT group, and the 43 remaining participants (mean age 29.6, SD 8.32 years; n=28, 65% female) were assigned to the waiting group. The 1-way ANOVA revealed no significant differences in age across the 3 groups *(F*_2, 198_=2.91; *P*=.06; ηp^2^=0.03). Similarly, no significant differences were found in the baseline SIAS scores (*F*_2, 198_=0.39; *P*=.68; ηp^2^<0.01), the baseline SPS scores (*F*_2, 198_=0.14; *P*=.87; ηp^2^<0.01), the baseline ESS scores (*F*_2, 198_=0.58; *P*=.56; ηp^2^<0.01), or the baseline BDI scores (*F*_2, 198_=0.90; *P*=.41; ηp^2^<0.01). The *χ^2^* test also indicated no significant differences in gender among the 3 groups (*χ*^*2*^_2_=1.519; *P*=.47). After the 8-week intervention or waiting, 98 participants successfully completed at least 6 weeks of the program and submitted posttest measurements. The details of the pre- and posttest measurement scores are shown in Table 1.

**Table 1.** Pre- and posttest scores for the 3 groups.

	Shame WCBT^a^, mean (SD) score		Normal WCBT, mean (SD) score		Waiting group, mean (SD) score	
	Pre (n=78)	Post (n=37)	Pre (n=80)	Post (n=35)	Pre (n=43)	Post (n=26)
Experience of Shame Scale	50.00 (12.57)	34.32 (11.42)	51.21 (13.43)	45.89 (11.85)	52.60 (12.59)	53.77 (10.21)
Social Interaction Anxiety Scale	49.39 (9.73)	30.16 (9.14)	50.20 (11.69)	38.43 (10.03)	51.11 (8.61)	49.85 (9.50)
Social Phobia Scale	37.67 (14.32)	20.97 (10.79)	37.11 (14.11)	26.25 (12.86)	38.44 (10.78)	39.54 (10.98)
Beck Depression Inventory	19.41 (10.33)	11.78 (10.00)	18.06 (8.62)	13.06 (6.85)	20.35 (9.43)	22.77 (10.83)

a WCBT: web-based cognitive behavioral therapy.

### Repeated Measures ANOVA for Pre- and Posttest Scores in the 3 WCBT Groups

A repeated measures ANOVA was conducted to examine the differences in the scores on the SIAS, SPS, ESS, and BDI between the 3 groups before and after WCBT treatment. The analysis used group as the between-group variable, time point as the within-group variable, and all 97 participants’ scores on the 4 scales (SIAS, SPS, ESS, and BDI) simultaneously as the dependent variables. Age was used as a covariate because there was a significant difference in age between the 3 groups before the test.

**Figure 2.**
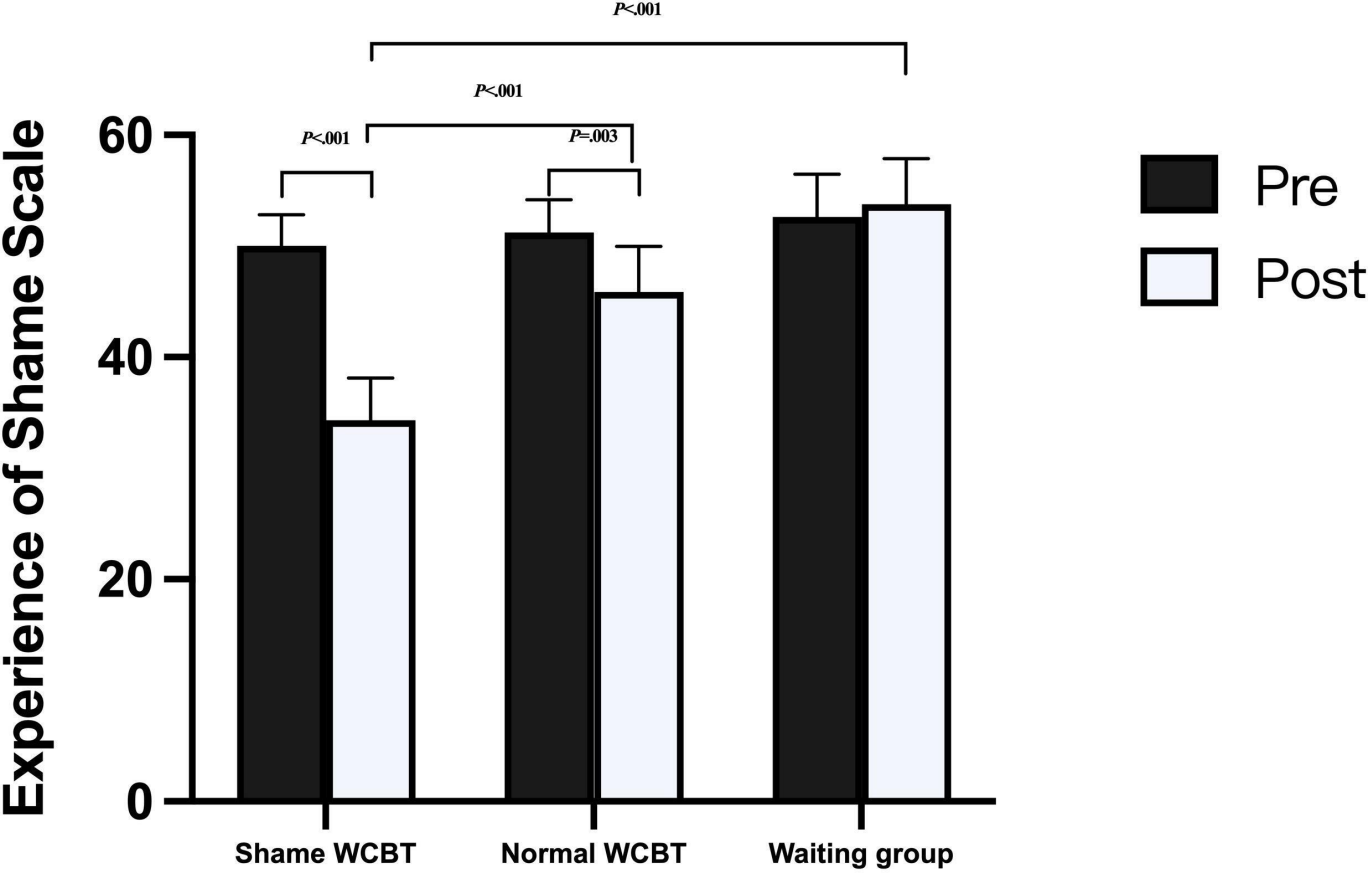
Experience of Shame Scale scores for the 3 groups. WCBT: web-based cognitive behavioral therapy.

For the ESS, the results indicated a significant interaction between time and group on the dependent variable BDI (*F*_2, 93_=13.40; *P*<.001; ηp^2^=0.22), and the time×age interaction was not significant (*F*_2, 93_=0.94; *P*=.34; ηp^2^=0.01). The interaction is shown in Figure 2. The main effect of time was significant (*F*_1, 93_=6.78; *P*=.01; ηp^2^=0.07), the main effect of group was significant (*F*_2, 93_=11.54; *P*<.001; ηp^2^=0.20), and the main effect of age was not significant (*F*_1, 93_=0.60; *P*=.44; ηp^2^=<0.01). Further simple effects analysis was conducted, and the difference in scores between the 2 comparisons of the 3 groups in the pretest scores was not significant (all *P* values were >.31). At the posttest, the difference in scores between the 3 groups was significant. The shame WCBT group scored significantly lower on the posttest than the waiting group (mean deviation −19.56; *P*<.001), the normal WCBT group scored marginally lower on the posttest than the waiting group (mean deviation −7.06; *P*=.06), and the shame WCBT group scored significantly lower than the normal WCBT group (mean deviation −12.50; *P*<.001).

The comparison of pre- and posttests in the 3 groups showed that the difference between the pre- and posttest scores in the shame WCBT group was significant (mean deviation −16.01; *P*<.001), and the difference between the pre- and posttest scores in the normal WCBT group was significant (mean deviation −6.06; *P*=.003). There was no significant difference in the waiting group (mean deviation −1.38; *P*=.54). These results indicate that patients with SAD in the shame intervention groups experienced significant reductions in shame levels (ESS) at the end of the intervention, and that the reduction was greater in the shame intervention group.

**Figure 3.**
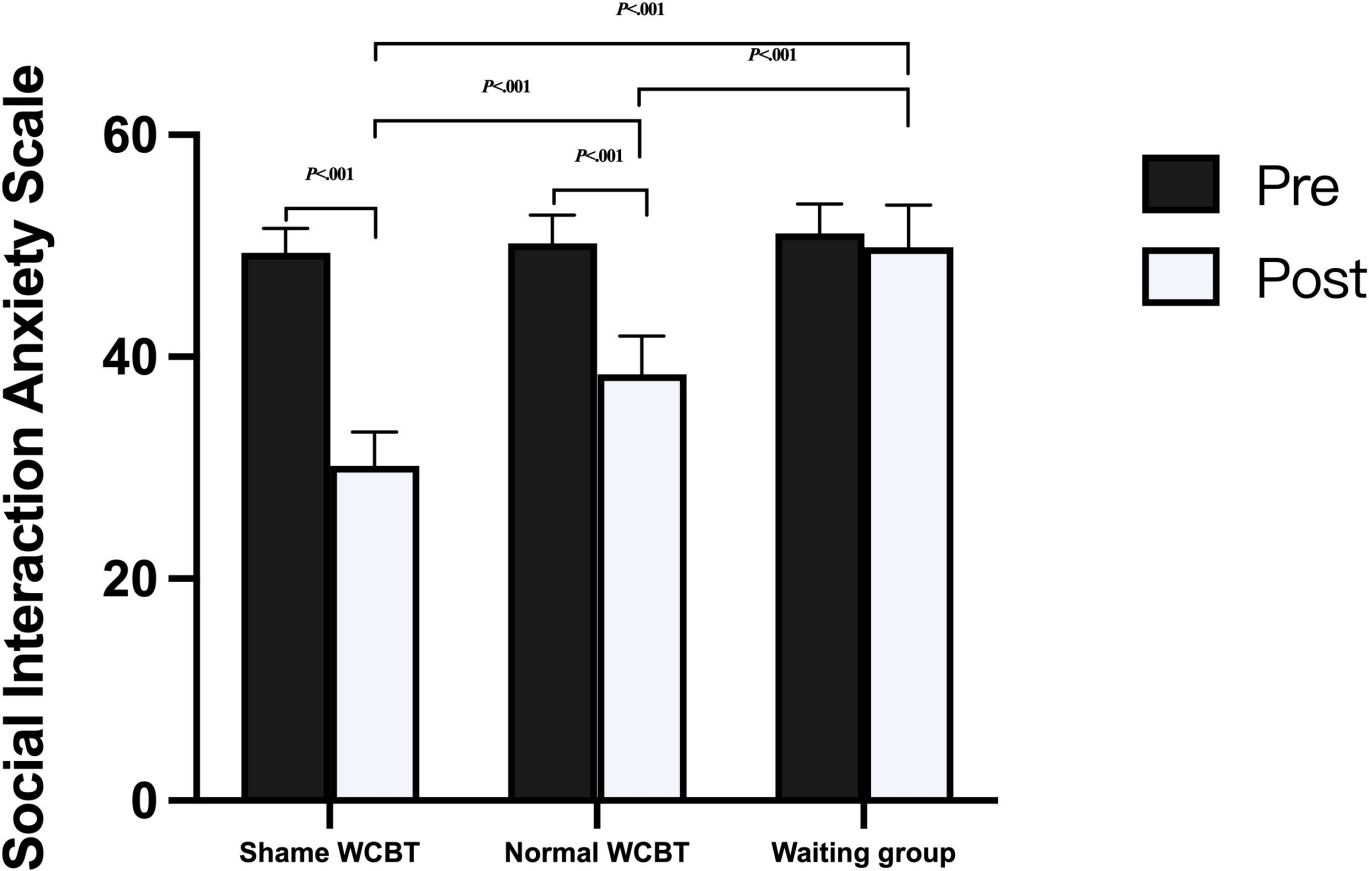
Social Interaction Anxiety Scale scores for the 3 groups. WCBT: web-based cognitive behavioral therapy*.*

For the SIAS, the results indicated a significant time×group interaction on the dependent variable SIAS (*F*_2, 93_=21.69; *P*<.001; ηp^2^=0.32), but the time×age interaction was not significant (*F*_2, 93_=0.11; *P*=.74; ηp^2^<0.01). The interaction is shown in Figure 3. The main effect of time was significant (*F*_1, 93_=6.99; *P*=.01; ηp^2^=0.07) the main effect of group was significant (*F*_2, 93_=16.81; *P*<.001; ηp^2^=0.27), and the main effect of age was significant (*F*_1, 93_=5.46; *P*=.02; ηp^2^=0.06). Further simple effects analysis was conducted, showing that the difference in scores between the 2 comparisons of the 3 groups in the pretest was not significant (all *P* values were >.40). At posttest, the difference in scores between the 3 groups was significant; the shame WCBT group scored significantly lower on the posttest than the waiting group (mean deviation −20.70; *P*<.001), the normal WCBT group scored significantly lower on the posttest than the waiting group (mean deviation −11.12; *P*<.001), and the shame WCBT group scored significantly lower than the normal WCBT group (mean deviation −9.58; *P*<.001).

The results of the comparison of pre- and posttests within the 3 groups showed that the difference between the pre- and posttest scores in the shame WCBT group was significant (mean deviation 20.07; *P*<.001), and the difference between the pre- and posttest scores in the normal WCBT group was significant (mean deviation 13.49; *P*<.001). There was no significant difference in the waiting group (mean deviation 3.07; *P*=.12). These results indicate that patients with SAD in both the normal and shame intervention groups experienced significant reductions in social anxiety levels (SIAS) at the end of the intervention and that the shame intervention group showed a higher reduction.

**Figure 4.**
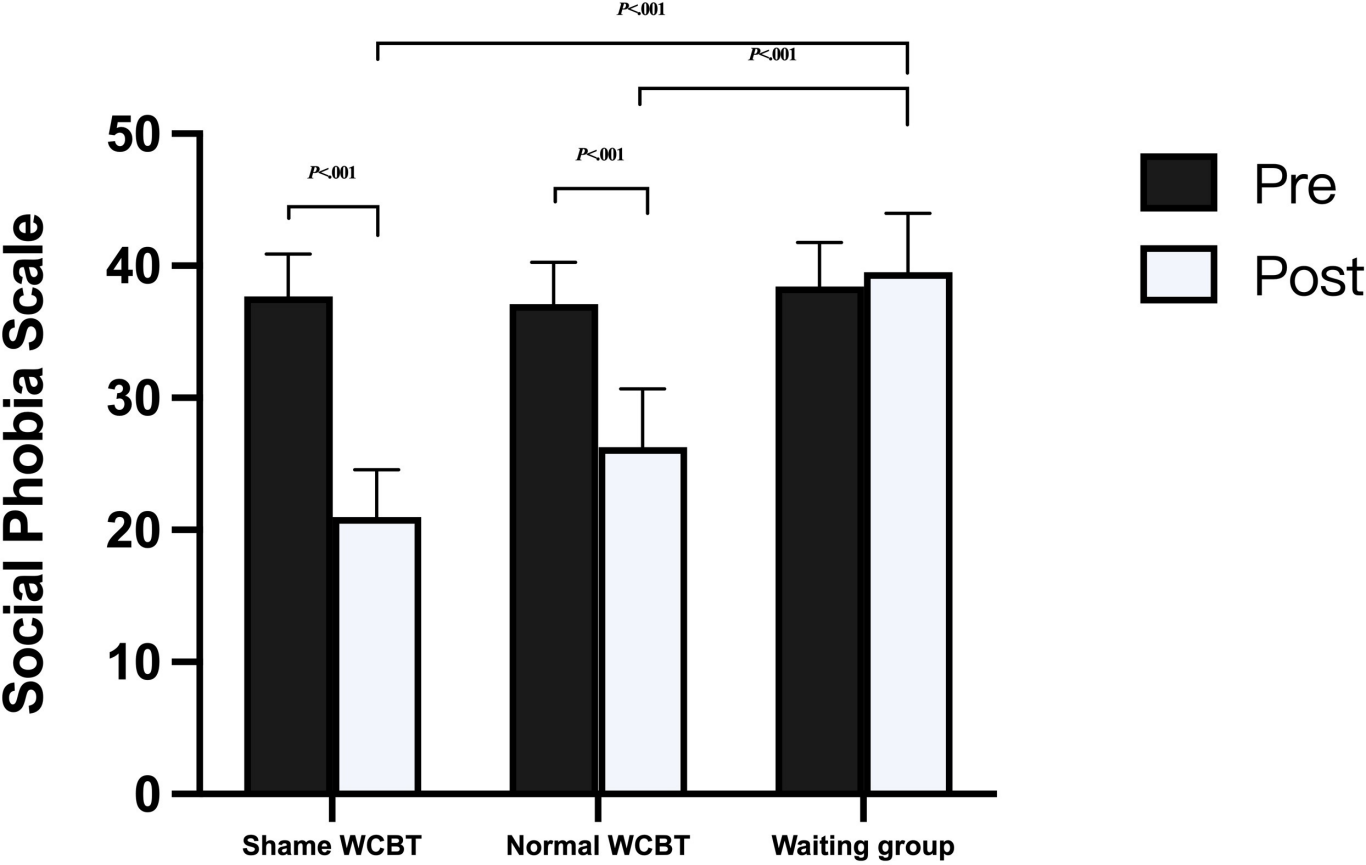
Social Phobia Scale scores for the 3 groups. WCBT: web-based cognitive behavioral therapy.

For the SPS, the results indicated a significant time×group interaction on the dependent variable SPS (*F*_2, 93_=10.93; *P*<.001; ηp^2^=0.19), and the time×age interaction was not significant (*F*_2, 93_=1.16; *P*=.28; ηp^2^=0.01). The interaction is shown in Figure 4. The main effect of time was significant (*F*_1, 93_=6.67; *P*=.01; ηp^2^=.07), the main effect of group was significant (*F*_2, 93_=6.81; *P*<.001; ηp^2^=0.13), and the main effect of age was not significant (*F*_1, 93_=1.03; *P*=.31; ηp^2^=0.01). Further simple effects analysis was conducted, and the difference in scores between the 2 comparisons of the 3 groups at pretest was not significant (all *P* values were>.99). At posttest, the difference in scores between the 3 groups was significant; the shame WCBT group scored significantly lower on the posttest than the waiting group (mean deviation −18.70; *P*<.001), the normal WCBT group scored significantly lower on the posttest than the waiting group (mean deviation −13.43; *P*<.001), and the shame WCBT group did not score significantly lower than the normal WCBT group (mean deviation −5.27; *P*=.20).

The results of the comparison of pre- and posttests within the 3 groups showed that there was a significant difference between the pre- and posttest scores in the shame WCBT group (mean deviation 17.12; *P*<.001) and the difference between the pre- and posttest scores in the normal WCBT group was significant (mean deviation 11.65; *P*<.001). There was no significant difference in the waiting group (mean deviation 0.52; *P*=.86). These results indicate that patients with SAD in both the normal and shame intervention groups experienced significant reductions in social phobia levels (SPS) at the end of the intervention and that there was no difference in the effects of the 2 interventions.

For the BDI scale, the results indicated a significant time×group interaction on the dependent variable BDI (*F*_2, 93_=6.90; *P*=.002; ηp2=0.13) and that the time×age interaction was not significant (*F*_2, 93_=0.20; *P*=.17; ηp^2^=0.02). The main effect of time was significant (*F*_1, 93_=6.18; *P*=.02; ηp^2^=0.06), the main effect of group was significant (*F*_2, 93_=6.37; *P*=.003; ηp^2^=0.12), and the main effect of age was not significant (*F*_1, 93_=0.18*; P*=.67; ηp^2^=<0.01). Further simple effects analysis was conducted, and the difference in scores between the 2 comparisons of the 3 groups in the pretest was not significant (all *P* values were >.80). At posttest, the difference in scores between the 3 groups was significant; the shame WCBT group scored significantly lower on the posttest than the waiting group (mean deviation −10.46; *P*<.001), the normal WCBT group scored significantly lower on the posttest than the waiting group (mean deviation −10.26; *P*<.001), and the shame WCBT group did not score significantly lower than the normal WCBT group (mean deviation −0.02; *P*>.99).

The results of the comparison of pre- and posttests within the 3 groups showed that the difference between the pre- and posttest scores in the shame WCBT group was significant (mean deviation −7.14; *P*<.001) and that the difference between the pre- and posttest scores in the normal WCBT group was significant (mean deviation −7.54; *P*<.001). There was no significant difference in the waiting group (mean deviation 0.87; *P*=.65). These results indicate that patients with SAD in both the normal and shame intervention groups experienced significant reductions in depression levels (BDI) at the end of the intervention and that there was no difference in the effects of the 2 interventions.

### Demographic Information and Mediation Analysis of Shame Intervention Design

**Figure 5.**
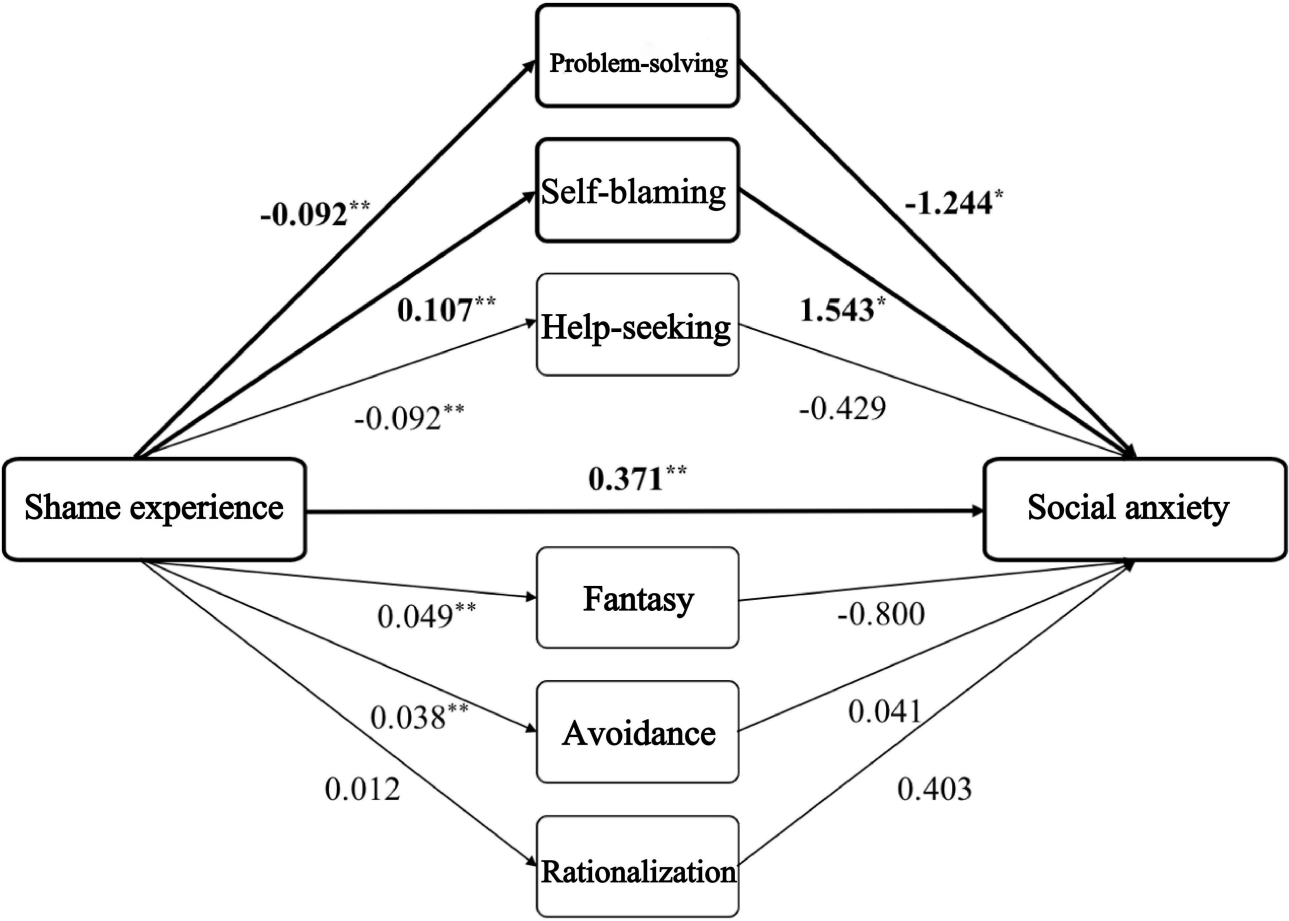
The coefficients and significance of coping style pathway. *P<.05 and **P<.01.

A total of 136 longitudinal questionnaires were received (with 153 questionnaires collected at pretest); 107 (69%) participants who met the criteria for high social anxiety (SIAS) were screened for final data processing (mean age 23.67, SD 4.497 years; n=55 female). Their mean ESS score was 64.08 (SD 17.55) and mean SIAS score was 36.17 (SD 17.42). The mediation analysis focused on the 6 coping style variables (problem-solving, self-blame, help-seeking, fantasy, avoidance, and rationalization) as potential mediators between shame experience and social interaction anxiety (SIAS). The absolute values of the correlation coefficients of all independent variables for 2-by-2 comparisons were less than 0.7, indicating the absence of multicollinearity. The coefficients and significance results of each pathway are shown in Figure 5.

When examining the results of the 6 mediating variables simultaneously, problem-solving and self-blame mediated the effect between shame experience and social interaction anxiety (SIAS). The 95% CI for problem-solving as a mediating variable was 0.025-0.217 and the indirect effect of the mediating variable was 0.115 (SE 0.049); the 95% CI for self-blame as a mediating variable was 0.024-0.339 and the indirect effect of the mediating variable was 0.165 (SE 0.082). Help-seeking, fantasy, avoidance, and rationalization were not significant mediating effects when used as mediating variables. The direct effect of shame experience on social interaction anxiety (SIAS) was 0.371 (SE 0.110; *t*_96_=3.372*; P*=.003; *R*^2^=0.26).

## Discussion

This is the first study to design and incorporate a shame intervention component in WCBT and conduct a randomized controlled trial to validate the efficacy of the shame WCBT. Based on a comparison of the ESS scores, the shame WCBT group showed a significant reduction in shame level after treatment compared to the normal WCBT and waiting groups. The reduction of shame level in participants with social anxiety is in line with the treatment response of other CBT strategies with shame intervention [13,14]. Additionally, both the shame WCBT group and the normal WCBT group showed a significant reduction in social anxiety symptoms (SIAS and SPS scores) after receiving the 8-week WCBT intervention, while participants in the shame intervention group showed a more rapid reduction in SIAS scores. These results highlight the effectiveness of WCBT in alleviating their symptoms of social anxiety. Furthermore, the shame WCBT intervention was significantly more effective for SAD than the waiting control condition, and it prompted a more significant reduction in social interaction–related anxiety and shame experience than normal WCBT. This suggests that the shame WCBT had significant intervention effects for patients with SAD [18,20,21] and that the shame intervention component could enhance the efficacy of normal WCBT.

The design of the shame intervention component was derived from longitudinal data and the results of previous shame studies [4-7]. In the Chinese cultural context, there is a strong and significant correlation between shame and social anxiety [7,13,24]. One interesting finding is that problem-solving and self-blaming partially mediated the effect of shame on social anxiety. Specifically, the stronger the shame experience and the more negative the attitude toward problem-solving, the more likely the individual was to think and act in a self-blaming manner when faced with social difficulties and less likely to adopt the corresponding behavior. Consequently, social anxiety symptoms have been shown to intensify [41,42]. The positive effect of the shame intervention could be understood as follows: first, the new content concerning psychological education on shame helped participants to comprehend the significant positive association between shame and social anxiety symptoms [24]. Second, according to a previous study, self-blame leads to a worsening of social anxiety symptoms [41], while problem-solving is a specific mediating variable between shame and social anxiety in Chinese people [42]. After the shame WCBT, participants adjusted inward their attribution of shame and consolidated it with active coping with shame through cognition reconstruction and exposure. Therefore, this study supports previous findings that explicit intervention targeting shame could enhance the efficacy of CBT [9].

One interesting finding is the outcome with respect to the SPS, which is contrary to previous studies: the effects of the shame intervention were not significantly different from the normal WCBT intervention group. This difference may be attributed to the fact that the SIAS and SPS measure similar but different aspects. Although both scales showed a high correlation between the scores of socially anxious individuals [32], the focus of the 2 scales remains different. The SIAS focuses on individuals’ anxiety in general social situations, and shame is more related to interpersonal interaction situations and tends to accompany social situations to avoid more experiences of social rejection [43]. However, the SPS focuses on anxiety levels when participants are observed or watched by others in different situations [31]. The significant changes in SPS scores after both WCBT interventions compared to the waiting group suggest the efficacy of WCBT, while the nonsignificant change in SPS score between the 2 different WCBTs may suggest that participants still worry about being evaluated in situations where they are observed [44].

It is noteworthy that there was no significant difference between the shame WCBT and the previous WCBT with respect to the reduction in depression levels. This might imply that the shame WCBT intervention did not specifically target depression. The reduction in depression levels in both WCBT groups is in agreement with previous findings that the alleviation of social anxiety levels can also, to a certain extent, contribute to the improvement of depression [6].

This study set out to develop the first shame-specific WCBT intervention for social anxiety and aimed to reduce social anxiety levels by psychoeducation and increasing individual initiative in problem-solving and reducing self-blame. The results show that the shame intervention was consistent with the traditional intervention on all indicators of social anxiety, shame, and depression. Further, it was more effective than the traditional WCBT program with respect to social anxiety in general situations, and participants who underwent the shame intervention also showed lower levels of shame. These results suggest that it is essential to explore shame-based WCBT for social anxiety; shame interventions could provide a deeper understanding of SAD [9].

There are some limitations and further directions that should be taken into consideration. First, the study did not examine changes in clinical outcomes over time of the 2 WCBT interventions. Although participants were screened with structured clinical interviews, the study relied on self-reported measures. This omission limits our understanding of long-term clinical changes, such as whether the participants made better use of problem-solving strategies to cope with shame, potentially leading to long-term benefits in managing SAD. Second, the relationship between shame and social anxiety is more prominent in Eastern cultures. Therefore, it is not known whether WCBT with a shame intervention in a Western cultural framework would yield better outcomes. It is also noteworthy that the dropout rate for the shame group was not significantly higher than that for the normal group, and adherence was comparable to a previous WCBT adherence study in an Eastern culture (for SAD, the dropout rate was 53.1%) [22]. While high dropout rates in WCBT studies are not unusual [45], they pose challenges to the reliability and validity of our findings. The shame intervention in this study primarily focused on cognitive reconstruction and exposure, aligning with the established WCBT framework. Future research could consider incorporating a broader range of therapeutic approaches to potentially enhance treatment adherence and reduce dropout rates.

## Supplementary material

10.2196/50535 Checklist 1 CONSORT-EHEALTH (Consolidated Standards of Reporting Trials of Electronic and Mobile Health Applications and Online Telehealth; V 1.6.1)

## Abbreviations

BDI: Beck Depression Inventory
CBGT: cognitive behavioral group therapy
CBT: cognitive behavioral therapy
CSQ: Coping Styles Questionnaire
ESS: Experience of Shame Scale
MINI: Mini-International Neuropsychiatric Interview
SAD: social anxiety disorder
SIAS: Social Interaction Anxiety Scale
SPS: Social Phobia Scale
WCBT: web-based cognitive behavioral therapy
